# Advances in green nanobiotechnology: Data for synthesis and characterization of silver nanoparticles from ethanolic extracts of fruits and leaves of *Annona muricata*

**DOI:** 10.1016/j.dib.2019.104194

**Published:** 2019-06-25

**Authors:** Yahaya Gavamukulya, Hany A. El-Shemy, Amos M. Meroka, Edwin S. Madivoli, Esther N. Maina, Fred Wamunyokoli, Gabriel Magoma

**Affiliations:** a Department of Molecular Biology and Biotechnology, Pan African University Institute for Basic Sciences, Technology and Innovation (PAUSTI), P. O. Box, 62000-00200 Nairobi, Kenya; b Department of Biochemistry and Molecular Biology, Faculty of Health Sciences, Busitema University, P.O. Box, 1460 Mbale, Uganda; c Department of Biochemistry, Faculty of Agriculture, Cairo University, 12613 Giza, Egypt; d Department of Biochemistry, College of Health Sciences, University of Nairobi, P.O. Box 30197-00100 Nairobi, Kenya; e Department of Biochemistry, School of Medicine and Health Sciences, Kenya Methodist University, P.O. Box 267-60200 Meru, Kenya; f Department of Chemistry, College of Pure and Applied Sciences, Jomo Kenyatta University of Agriculture and Technology, P. O. Box, 62000-00200 Nairobi, Kenya; g Department of Biochemistry, College of Health Sciences, Jomo Kenyatta University of Agriculture and Technology, P. O. Box, 62000-00200 Nairobi, Kenya

**Keywords:** Annona muricata, Green synthesis, Silver nanoparticles (AgNPs), UV/VIS, FTIR, XRD

## Abstract

In this data article, data obtained from an efficient, eco-friendly and low-cost method for the synthesis and recovery of Silver nanoparticles (AgNPs) using ethanolic extracts of Annona muricata fruits and leaves as reducing, stabilizing and capping agents has been reported. 99.7% pure silver nitrate was used as the inorganic ion source. The data was obtained using different spectroscopic and microscopic techniques. The data is presented in form of images, Microsoft excel sheets, graphs,.raw files,.dpt files, PDF files, among others. Methods of analysis and interpretation of the data have also been presented. The data can be most useful to researchers, research students, industrialists and academicians to acquire knowledge on the green synthesis of AgNPs and related applications. The data is deposited in the Mendeley Data Repository as two independent datasets accessible at https://doi.org/10.17632/jkj2x782wh.1 Gavamukulya et al., 2019 and https://doi.org/10.17632/f4mb6b488n.1 Gavamukulya et al., 2019.

**Table:** Specifications table

Subject area	*Chemistry, Physics, Biotechnology, Materials Science*
More specific subject area	*Green chemistry, Nanobiotechnology*
Type of data	*Excel files, Tables, Data files, images, text files, graphs, PDF files*
How data was acquired	Ultraviolet Visible Spectrum *(UV/VIS) (Jenway Model* 6800 *Spectrophotometer Flight Deck****),*** Fourier Transform Infrared Resorption *(FTIR) (*Bruker Tensor II FT-IR spectrophotometer model (Bruker, Ettlingen, Germany)*,* X-Ray Diffraction Analysis *(XRD)* (D8 Advance; Bruker Optik, Ettlingen, Germany)*,*
Data format	*Raw, filtered*
Experimental factors	*Most of the analyses were conducted at room temperature*
Experimental features	*Samples were harvested, dried, milled and extracted with ethanol to obtain the ethanolic extracts. Extracts were then used for the synthesis via AgNO*_*3*_*solution. Prior to further characterization, the synthesized AgNPs were lyophilized. Further preparation of the samples was as reported for each protocol.*
Data source location	*Kaliro, Mbale, and Iganga (Uganda); Nairobi (Kenya); Fribourg (Switzerland)*
Data accessibility	*All datasets have been deposited in the public repository, Mendeley Data and is accessed via the following two links:*https://doi.org/10.17632/jkj2x782wh.1 and https://doi.org/10.17632/f4mb6b488n.1

**Table:** 

**Value of the data** - The data can be used to understand the UV/VIS absorption spectra including for storage, pH, and temperature stability, understand XRD patterns as well as FTIR spectra for the synthesized AgNPs. - There is no previous reported data related to the synthesis of AgNPs using ethanolic extracts of Annona muricata. - The data presents a new cost-effective process for recovery of the synthesized AgNPs. - The data can be most useful for researchers, research students, industrialists and academicians to acquire knowledge on the green synthesis of AgNPs. - Through replicating the protocols presented, the data can be helpful in development of other experiments in the field of green synthesis. - The data could be highlighted for a wider array of biomedical and clinical applications such as anticancer, antidiabetic, and anti-hypotensive uses among others.

## 1 Data

This data was obtained from the synthesis and characterization of highly stable silver nanoparticles from ethanolic extracts of fruits and leaves of *Annona muricata*. Because of the previously reported unique activities of extracts from *Annona muricata* [3], preparation of AgNPs from this plant especially with ethanolic extracts potentially offers unique advantages in comparison with those from other plant materials. The data described herein is composed of images, Microsoft excel data sheets, tables, among others. The following data is presented:

### 1.1 Data for synthesis and characterization of AgNPs from ethanolic extracts of fruits (AgNPs-F) of *Annona muricata*[1]

a. Images of the formed AgNPs for AgNPs-F.
b. Data for UV/VIS at 72 hours for AgNPs-F
c. Consolidated data for each of the pH values studied for stability (2, 4, 7, 9, 11) for AgNPs-F
d. Consolidated data for the temperature values studied for stability (25 °C, 35 °C, 45 °C, 55 °C, 65 °C, 75 °C, and 85 °C) for AgNPs-F
e. Consolidated data for the storage temperature values studied for stability at 3 months (25 °C, 4 °C, −20 °C and −80 °C) for AgNPs-F
f. Raw data for the FTIR Results for AgNPs-F
g. Raw data for XRD as well as an analysis report showing how the data was analyzed for AgNPs-F

### 1.2 Data for synthesis and characterization of AgNPs from ethanolic extracts of leaves (AgNPs-L) of *Annona muricata*[2]

h. Images of the formed AgNPs for AgNPs-L.
i. Data for UV/VIS at 72 hours for AgNPs-L
j. Consolidated data for each of the pH values studied for stability (2, 4, 7, 9, 11) for AgNPs-L
k. Consolidated data for the temperature values studied for stability (25 °C, 35 °C, 45 °C, 55 °C, 65 °C, 75 °C, and 85 °C) for AgNPs-L
l. Consolidated data for the storage temperature values studied for stability at 3 months (25 °C, 4 °C, −20 °C and −80 °C) for AgNPs-L
m. Raw data for the FTIR Results for AgNPs-L
n. Raw data for XRD as well as an analysis report showing how the data was analyzed for AgNPs-L

## 2 Experimental design, materials, and methods

### 2.1 Plant material collection and authentication

Fresh leaves and ripe fruits of *Annona muricata* were collected from the wild in Eastern Uganda in the districts of Kaliro, Iganga and Mbale during the month of January 2018. The plant was identified and authenticated in the Makerere University Botanical Herbarium (MHU) by Dr Namaganda Mary and a voucher specimen was deposited in the herbarium with the accession number MHU50860.

### 2.2 Plant material preparation and drying

The Fruits of *Annona muricata* were washed with clean water and then peeled to remove the fresh pulp. The pulp was then cut into small pieces and placed in a hot air oven to dry at 50 °C for a week. The dried pulp was then milled into a powder using an electric grater and then kept at 4 °C until use.

The leaves of *Annona muricata* were washed with clean water and cut into small pieces, dried at room temperature and then powdered in an electric grater and kept at 4 °C until use.

### 2.3 Sample preparation for green synthesis

700 g of powdered fruits were extracted using 2000 ml of absolute ethanol for three days by the plant tissue homogenization method as previously described [4]. The light brown Ethanolic Extracts of *Annona muricata* fruits were then filtered and kept at 4 °C until use.

700 g of powdered leaves were extracted using 2000 ml of absolute ethanol for three days by the plant tissue homogenization method as previously described [4]. The dark green Ethanolic Extracts of *Annona muricata* leaves were then filtered and kept at 4 °C until use.

### 2.4 Preparation of the 1mM AgNO_3_ solution

Extra pure AgNO_3_ (99.7% purity) was used for the preparation of the AgNO_3_ solution. 0.1699g of AgNO_3_ were weighed on an ultrasensitive measuring balance and transferred to 1000ml volumetric flask. Distilled water was then added to the volumetric flask with continuous shaking until the 1000ml mark was reached. The solution was then left to completely dissolve the salt. The 1mM AgNO_3_ solution had been successfully prepared.

### 2.5 Synthesis of silver nanoparticles from fruits extracts (AgNPs-F)

The AgNPs were synthesized as previously described [5], [6], [7], [8]. For each run, about 50 ml of the filtered fruits extract was mixed with about 450 ml of 1 mM AgNO_3_ solution in a 500ml flask and mixed thoroughly, forming a uniform mixture. The mixture was then rested at room temperature in the dark storage cabinets for up to about 72 hours, with continuous monitoring. After about 3hours, the mixture was observed to start changing from light brown to yellowish brown. After about 72 hours, the mixture had completely changed colour to dark brown. This color change is visual evidence of formation of AgNPs or reduction of silver ions into AgNPs due to the excitation of surface plasmon vibration [6], [8], [9], [10].

### 2.6 Synthesis of silver nanoparticles from leaves extracts (AgNPs-L)

AgNPs were synthesized by the following method. For each run, about 50 ml of the filtered leaves extract was mixed with about 450 ml of 1 mM AgNO_3_ solution in a 500ml flask and mixed thoroughly, forming a uniform mixture. The mixture was then rested at room temperature in the dark storage cabinets for up to about 72 hours, with continuous monitoring. After about 3hours, the mixture was observed to start changing from dark green to yellowish brown. After about 72 hours, the mixture had completely changed colour to dark brown. This color change is visual evidence of formation of AgNPs or reduction of silver ions into AgNPs due to the excitation of surface plasmon vibration [6], [8], [9], [10].

### 2.7 Characterization of the AgNPs

The green synthesized AgNPs were characterized using UV/VIS, XRD and FTIR techniques as previously described [5], [6], [7]. This characterization was aimed at elucidating the absorption maxima, size, shape, heat stability among other parameters of the AgNPs.

### 2.8 UV/VIS measurements to confirm formation of AgNPs

The synthesis of AgNPs from the ethanolic extract of fruits and leaves of *Annona muricata* was further confirmed by ultraviolet–visible spectroscopy (UV/VIS) in the range of between 300nm and 650nm [6], [8], [9], [11] and ethanol was used as a blank.

### 2.9 Temperature/heat stability of the synthesized AgNPs

About 10ml of the formed AgNPs suspension in boiling tubes were subjected to different temperature conditions by heating in a digital water bath for about 3 minutes each and measuring the absorbance spectra on the UV/VIS in a scan range of 350nm–650nm [12]. The temperature tested included room temperature (25 °C), 35 °C, 45 °C, 55 °C, 65 °C, 75 °C, and 85 °C.

### 2.10 pH stability of the synthesized AgNPs

About 15ml of the formed AgNPs suspension was aliquoted into 5 test tubes each containing about 3 ml of the AgNPs suspension. The suspensions in the test tubes were then adjusted to and subjected to different pH conditions ranging from about pH 2 to about pH 11. The suspension in each test tube was subjected to a different pH condition. The specific pH conditions tested were pH 2, 4, 7, 9, and 11. The pH were adjusted by either adding drops of 1N NaOH or 1N HCl until the desired pH was achieved as observed on the pH meter [12], [13]. The absorbance spectra of the suspensions were then measured on the UV/VIS in a scan range of 300nm–650nm.

### 2.11 Storage stability of the AgNPs

About 20ml of the formed AgNPs suspension was aliquoted into four 15ml universal tubes each containing about 5 ml of the AgNPs suspension. The suspensions in the tubes were then stored at different temperature conditions for a period of 3 months. The temperatures at which the storage was done included room temperature (which varied between at about 20 °C–30 °C during the experimental period), 4 °C, −20 °C and −80 °C. At the end of the 3 months, the samples were retrieved from the different storage facilities allowed to thaw at room temperature and then their absorbance spectra were measured on the UV/VIS in a scan range of 300nm–650nm.

### 2.12 Recovery of the synthesized AgNPs

The AgNPs suspension were recovered by freezing, centrifugation and freeze drying.

### 2.13 Functional groups analysis using FTIR

FTIR measurements were carried out to identify the promising biomolecules in the *Annona muricata* ethanolic extract accountable for the reduction of the silver ions and also the capping agents liable for the stability of the bio-reduced AgNPs. The functional groups present in the AgNPs were analyzed by a Bruker Tensor II FT-IR spectrophotometer model (Bruker, Ettlingen, Germany). The KBr pellets of samples were prepared by grinding 10 mg of samples, with 250 mg KBr (FT-IR grade). The 13 mm KBr pellets were prepared in a standard device under a pressure of 75 kN cm^−2^ for 3 min. The spectral resolution was set at 4 cm^−1^ and the scanning range from 400 to 4000 cm^−1^ [14]. The representative FTIR spectra of the recovered and dried AgNPs synthesized from ethanolic extracts of fruits of *Annona muricata* were recorded and the major and minor peaks were manifested and identified accordingly.

### 2.14 Crystalline size determination using XRD

XRD analysis was employed to determine the average crystalline size of the AgNPs formed. The XRD (D8 Advance; Bruker Optik, Ettlingen, Germany) with CuKα radiation (λ = 1.5406 Å) and working at 40 kV/40 mA in the range of 10°–80° with a 2°-per-minute scanning rate was used. The XRD diffraction data was analyzed using the Match! Software (Crystal Impact, Bonn, Germany) and the average crystalline size of the AgNPs formed in the bio-reduction was determined using the Scherrer equation, with a constant of 0.94.
